# High-fat diet-induced obesity and impairment of brain neurotransmitter pool

**DOI:** 10.1515/tnsci-2020-0099

**Published:** 2020-06-01

**Authors:** Ranyah Shaker M. Labban, Hanan Alfawaz, Ahmed T. Almnaizel, Wail M. Hassan, Ramesa Shafi Bhat, Nadine MS Moubayed, Geir Bjørklund, Afaf El-Ansary

**Affiliations:** Department of Food Science and Nutrition, College of Food and Agriculture Sciences, King Saud University, Riyadh, Saudi Arabia; Ministry of Health, General Administration of Nutrition, Riyadh, Saudi Arabia; Department of Food Science and Nutrition, College of Food and Agriculture Sciences, King Saud University, Riyadh, Saudi Arabia; Prince Naif for Health Research Center, King Saud University, Riyadh, Saudi Arabia; Department of Biomedical Sciences, University of Missouri-Kansas City School of Medicine, Missouri, USA; Biochemistry Department, Science College, King Saud University, Riyadh, Saudi Arabia; Botany and Microbiology Department, College of Sciences, King Saud University, Riyadh, Saudi Arabia; Council for Nutritional and Environmental Medicine, Mo i Rana, Norway; Central Laboratory, Female Centre for Scientific and Medical Studies, King Saud University, Riyadh, Saudi Arabia

**Keywords:** dyslipidemia, gut microbiota, inflammation, obesity, oxidative stress, synaptopathy

## Abstract

Obesity and the brain are linked since the brain can control the weight of the body through its neurotransmitters. The aim of the present study was to investigate the effect of high-fat diet (HFD)-induced obesity on brain functioning through the measurement of brain glutamate, dopamine, and serotonin metabolic pools. In the present study, two groups of rats served as subjects. Group 1 was fed a normal diet and named as the lean group. Group 2 was fed an HFD for 4 weeks and named as the obese group. Markers of oxidative stress (malondialdehyde, glutathione, glutathione-*s*-transferase, and vitamin C), inflammatory cytokines (interleukin [IL]-6 and IL-12), and leptin along with a lipid profile (cholesterol, triglycerides, high-density lipoprotein, and low-density lipoprotein levels) were measured in the serum. Neurotransmitters dopamine, serotonin, and glutamate were measured in brain tissue. Fecal samples were collected for observing changes in gut flora. In brain tissue, significantly high levels of dopamine and glutamate as well as significantly low levels of serotonin were found in the obese group compared to those in the lean group (*P* > 0.001) and were discussed in relation to the biochemical profile in the serum. It was also noted that the HFD affected bacterial gut composition in comparison to the control group with gram-positive cocci dominance in the control group compared to obese. The results of the present study confirm that obesity is linked to inflammation, oxidative stress, dyslipidemic processes, and altered brain neurotransmitter levels that can cause obesity-related neuropsychiatric complications.

## 1 Introduction

It is unquestionable that obesity continues to increase worldwide. Recently, the prevalence of obesity was found to be threefold higher than in the last 40 years. In 2018, the World Health Organization (WHO) reported that globally 39% of the adults are overweight (body mass index [BMI] ≥ 25 kg/m^2^) and 13% are obese (BMI ≥ 30 kg/m^2^) [1]. Research has shown that obesity increases the risk of developing metabolic illnesses [1,2] and disturbs brain structure and function [3,4,5]. For instance, it is known that regional cerebral blood flow is decreased in individuals with obesity, particularly in prefrontal brain regions that are involved in cognitive, attention, and decision-making functions [6]. In addition, obesity is associated with impaired gray and white matter due to inflammation [7]. For most neurotransmitters, only a small amount of the total stored pool is released with each nerve stimulation; nonetheless, it was interesting to find that obesity is associated with brain-level molecular changes [8].

Dietary forms, mainly for those who prefer fat intake, often have been responsible for the increase in body weight and adiposity [9]. High-fat diets (HFDs) have been used to induce obesity in animals in a model first termed *nutritional obesity* and later renamed *dietary obesity* [10]. The hormone leptin plays an important role regarding intake of food, and the homeostasis of body weight is commonly increased in obesity, a condition known as *leptin resistance*. Hyperleptinemia is known to have a considerable role in the pathogenesis of obesity and obesity-related neurological conditions [11]. Prolonged presence of glutamate as an excitatory amino acid and reactive oxygen species (ROS) can induce excitotoxicity and oxidative stress, respectively, which are two of the major mechanisms responsible for neuronal damage [12]. Several pieces of evidence have pointed out that excitotoxicity and oxidative stress are related in that excitatory events may stimulate the formation of ROS, which can lead to the release of excitatory amino acids under any stressful condition. Glutamate receptor agonists are able to induce the formation of ROS. It is well documented that under normal conditions, leptin regulates glutamate *N*-methyl-d-aspartate receptors (NMDA-R) and provides neuroprotective effects on cells. However, obesity impairs this pathway, and an increase in brain glutamate levels leads to a dysfunction in extrasynaptic NMDA-R, a decrease in long-term potentiation, and mitochondrial dysfunctions. Thus, the slow excitotoxicity shown in Alzheimer’s disease due to overexcitation of NMDA receptors by glutamate can be linked to obesity through leptin resistance as a risk factor for neurological disorders [13,14].

The association between obesity and chronic low-grade inflammation has been well researched [15]. An increase in the circulatory concentrations of numerous cytokines can be observed in obese individuals [15]. Many of these proinflammatory molecules are secreted by adipocytes, and it is hypothesized that enlarged adipose tissue mass is either directly or indirectly associated with increased production of proinflammatory cytokines [16].

Significant increases in proinflammatory cytokines, including interleukin (IL)-6 and IL-12, were found in generally obese individuals compared to nonobese ones [17]. For cases of central obesity specifically, Schmidt et al. reported higher levels of IL-5, IL-6, IL-12, IL-13, and interferon-γ in participants with abdominal obesity compared to those without [17]. In another study, IL-10 was significantly increased, whereas tumor necrosis factor-alpha and IL-6 decreased, following calorie restriction [18]. Thus, the use of drugs or specific bioactive food components with anti-inflammatory properties may help to reduce the inflammatory state associated with obesity and overcome leptin resistance, especially at the hypothalamus.


**Ethics approval**: The research related to animal use has been complied with all the relevant national regulations and was approved by the Graduate Studies and Scientific Research Ethical Committee of Bioethics at King Saud University, for the care and use of animals.

## 2 Materials and methods

### 2.1 Animals and diet

**Figure 1:**
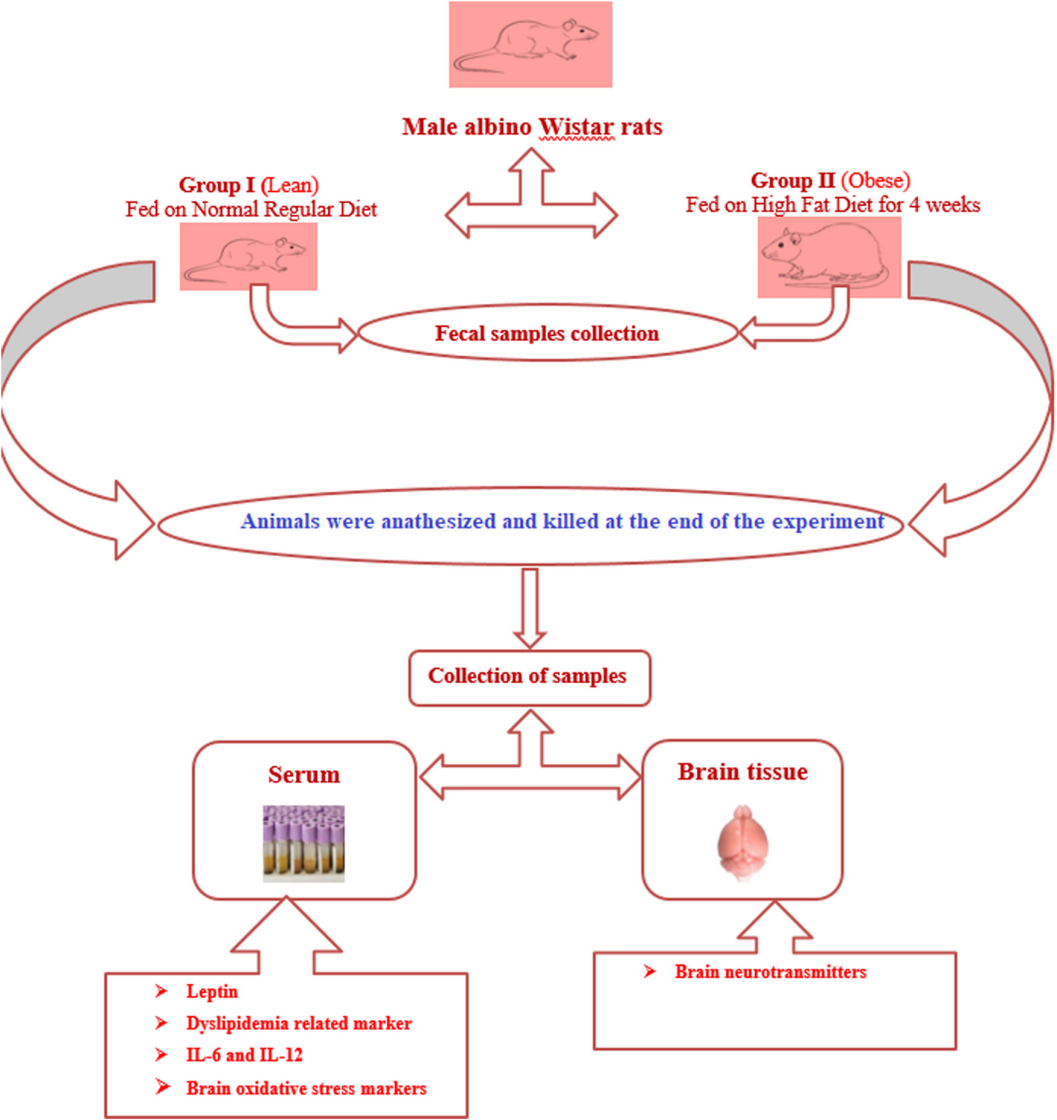
Flow chart of the study design and experimental procedure.

A predetermination of sample size calculation was not performed. Twelve male Wistar albino rats weighing 100 ± 20 g, at the age of 4 weeks, were housed individually in stainless steel cages under controlled environmental conditions of 25℃, 12 h day/night cycle, and a humidity of 50% ± 5. Rats were randomly and blindly classified into two groups of six rats each as follows: Group 1, the control group had free access to water and standard diet. Group 2, the obese group was fed with HFD (60% fats) for four consecutive weeks. Feeding of animals was done under sterilized controlled conditions at the Experimental and Surgery Animal Laboratory, King Khalid Hospital. TestDiet^®^ company formula was used to prepare HFD with the help of Prince Naif Health Research Center, King Khalid Hospital, College of Medicine, King Saud University and prepared diet was 60% rich in fat content. At the end of the experiment, animals were anesthetized with 5.0% of sevoflurane and 100% oxygen, and the flow rate of sevoflurane was determined by the following formula: flow rate (mL/min) = 0.5 × body weight (g). Blood and whole brain tissue samples were collected from all the animals under study. Fecal samples were also collected after 2 and 4 weeks from the start of the study from all the animals of each group. The experimental design is illustrated in Figure 1.

#### 2.1.1 Measurement of the BMI

Body length (nose–anus length) was determined for all anesthetized rats. The body weight and body length were used to determine the BMI using the following equation.

Body mass index (BMI) = Body weight (g)/body length^2^ (cm^2^).

#### 2.1.2 Collection of samples

Blood sample was collected by direct cardiac puncture in a plane tubes without anticoagulant. It was centrifuged at 1,100 × *g* for 10 min to separate blood serum. The collected serum samples were immediately stored at −80℃ until use. Whole brain was collected, washed, dissected to small pieces, and immediately stored at −80℃ deep freezer until further use. Feces were collected from both groups after 2 and 4 weeks.

#### 2.1.3 Preparation of brain tissue homogenates

After collection, the brain tissue was washed using cold normal saline and homogenized in ten volumes/weight of phosphate-buffered saline (PBS). Then, the homogenate was centrifuged at 1,500 × *g* for 10 min, and the clear supernatant was gathered and used for the measurements of neurotransmitters as described subsequently.

#### 2.1.4 Leptin and dyslipidemia-related markers

Leptin in serum was estimated with a quantitative sandwich ELISA kit from MyBioSource. Quantitative estimations of cholesterol, triglycerides, high-density lipoprotein (HDL), and low-density lipoprotein (LDL) were calculated with kits from United Diagnostics Industry.

#### 2.1.5 IL-6 and IL-12

The concentrations of IL-6 and IL-12 in the serum samples were determined using a quantitative sandwich enzyme ELISA kit from MyBioSource.

#### 2.1.6 Oxidative stress markers

The method of Ruiz-Larrea et al. [19] was used to determine lipid oxidation. An assay of vitamin C was performed according to the method of Jagota and Dani [20]. The method provided by Beutler et al. [21] was used to estimate glutathione (GSH) by utilizing 5,5′-dithiobis 2-nitrobenzoic acid combined with sulfhydryl compounds in order to obtain a relatively stable yellow color. Glutathione S-transferase (GST) activity was determined by the GST-catalyzed reaction between GST substrate, GSH, and 1-chloro-2,4-dinitrobenzene [22].

#### 2.1.7 Brain neurotransmitters

The concentrations of dopamine and glutamate were determined using a Competitive ELISA kit from MyBioSource. The concentration of serotonin was determined using the Quantitative Sandwich ELISA kit from MyBioSource.

#### 2.1.8 Microbiological analyses

##### 2.1.8.1 Fecal sample collection and preparation

After collection, fecal matter was homogenized as 1 g in 10 mL of 0.1 M, pH 7.2, PBS. The homogenate was centrifuged at 4,500 rpm and 4℃ for 3 min. Finally, 1 mL of the resulting fecal supernatant was serially diluted four times with a 9 mL solution of sterile PBS [23].

### 2.2 Bacterial enumeration and culturing

One hundred microliters of each prepared dilution were loaded onto the surface of different culture media, including MacConkey plates, nutrient agar (Oxoid) plates, and blood agar plates as well as cycloserine cefoxitin fructose agar (CCFA) plates, a selective medium to grow *Clostridium* bacteria. Anaerobic jars containing 5% CO_2_ at 37℃ were used for the CCFA plates with a 3 day incubation period. The incubation time for all other culture media was 18–24 h under aerobic conditions at 37℃. The experiment was repeated twice, and the average number of bacteria per plate was recorded each time. Gram staining and biochemical tests were used to identify the bacterial strains.

### 2.3 Statistical analysis

The results are expressed as mean ± SD. To statistically compare the results between groups, one-way analysis of variance tests were used. Significance was assigned at the level of *P* < 0.05. The receiver operating characteristics curve (ROC) analysis with the area under the curve (AUC), cutoff values, and the degrees of sensitivity and specificity were calculated. We used Pearson moment correlation coefficient and multiple regression analysis to determine the most predictive biomarkers of the three neurotransmitters, serotonin, dopamine, and glutamate. As a first step, we used Pearson correlation to test the correlation of each of the biomarkers individually with each of the neurotransmitters. Next, a multiple regression model was constructed using the stepwise method. Both Pearson moment correlation coefficient and multiple regression analysis were performed using IBM SPSS version 24 (IBM Corp., Armonk, NY, USA).

## 3 Results

In the present study, animals were fed an HFD composed of saturated fats in addition to coconut oil. Table 1 demonstrates mean ± SD of weight gain and BMI in the two studied groups. We found a significant difference between the obese and lean rats (*P* < 0.001). While the BMI in obese rats recorded a value of 0.86 ± 0.09 g/cm^2^, the normal weight group recorded a significantly lower value of 0.55 ± 0.04 g/cm^2^ (*P* < 0.001).

**Table 1:** Weight gain and BMI as measures of obesity in the two studied groups

Parameters	Groups	Min.	Max.	Mean ± SD	Percent change	*P* value^a^
Weight gain	Lean	120.00	199.00	152.00 ± 30.83	100.00	
	HFD-obese	425.00	513.00	458.60 ± 35.66	201.31	0.001
BMI	Lean	0.51	0.62	0.55 ± 0.04	100.00	
	HFD-obese	0.79	1.01	0.86 ± 0.09	156.36	0.001

^a^Refers to the significant difference between lean and HFD-induced obese rats.

Table 2 presents the brain levels of serotonin, dopamine, and glutamate in the two groups. It can be easily observed that compared with lean rats, serotonin was significantly lower, and dopamine and glutamate were significantly higher in obese rats compared to lean rats (*P* < 0.003, *P* < 0.001, and *P* < 0.001, respectively).

**Table 2:** Brain neurotransmitters as measures of synaptopathy in HFD-induced obese rats compared to lean rats

Parameters	Groups	Min.	Max.	Mean ± SD	Percent change	*P* value^a^
Serotonin (ng/mL)	Lean	67.00	100.00	77.40 ± 14.50	100.00	
	HFD-obese	34.00	43.00	39.00 ± 3.67	50.40	0.003
Dopamine (ng/mL)	Lean	23.00	49.00	34.60 ± 11.37	100.00	
	HFD-obese	72.00	105.00	92.40 ± 12.82	167.05	0.001
Glutamate (ng/mL)	Lean	22.00	44.00	36.20 ± 8.79	100.00	
	HFD-obese	55.00	71.00	64.80 ± 6.02	179.00	0.001

^a^Refers to the significant difference between lean and HFD-induced obese rats.

Table 3 demonstrates the IL-12 and IL-6 serum levels in the two studied groups. Both inflammatory cytokine levels were much lower in the serum of lean rats (44.14 ± 6.68 and 84.50 ± 6.22, respectively) compared to obese rats fed an HFD (149.11 ± 11.24 and 280.15 ± 8.42, respectively) (*P* < 0.001).

**Table 3:** IL-12 and IL-6 as measure of inflammation in HFD-induced obese compared to lean rats

Parameters	Groups	Min.	Max.	Mean ± SD	Percent change	*P* value^a^
IL-12 (pg/mL)	Lean	33.81	50.27	44.14 ± 6.68	100.00	
	HFD-obese	133.55	161.69	149.11 ± 11.24	237.81	0.001
IL-6 (pg/mL)	Lean	77.14	92.47	84.50 ± 6.22	100.00	
	HFD-obese	269.52	291.11	280.15 ± 8.42	231.36	0.001

^a^Refers to the significant difference between lean and HFD-induced obese rats

Table 4 demonstrates a significant increase in leptin in HFD-induced obese rats (*P* < 0.001).

**Table 4:** Leptin in HFD-induced obese rats compared to lean rats

Parameters	Groups	Min.	Max.	Mean ± SD	Percent change	*P* value^a^
Leptin (ng/mL)	Lean	0.71	1.09	0.89 ± 0.14	100.00	
	HFD-obese	3.39	3.57	3.48 ± 0.07	291.011	0.001

^a^Refers to the significant difference between lean and HFD-induced obese rats.

Table 5 demonstrates lipid peroxides (malondialdehyde [MDA]) as an oxidative stress marker together with GSH, GST, and vitamin C as markers of antioxidant capacity. It can be noticed that the four measured variables were nonsignificantly different between the two groups.

**Table 5:** Oxidative stress related markers in HFD-induced obese rats compared to lean rats

Parameters	Groups	Min.	Max.	Mean ± SD	Percent change	*P* value^a^
MDA	Lean	92.94	126.66	108.95 ± 12.54	100.00	
	HFD-obese	80.71	114.83	94.97 ± 13.70	87.25	0.131
GSH	Lean	103.38	185.99	148.31 ± 36.42	100.00	
	HFD-obese	154.10	203.86	168.50 ± 20.49	113.61	0.312
GST	Lean	0.94	1.88	1.35 ± 0.42	100.00	
	HFD-obese	0.52	4.17	1.64 ± 1.46	121.48	0.600
Vitamin C	Lean	0.03	0.05	0.04 ± 0.01	100.00	
	HFD-obese	0.02	0.03	0.03 ± 0.00	125.00	0.075

^a^Refers to the significant difference between lean and HFD-induced obese rats.

Tables 6 and 7 present the occurrence of dyslipidemia in the obese rats compared to lean rats. This irregularity presents as significantly higher cholesterol, higher triglycerides, remarkably higher LDL, nonsignificantly lower HDL, together with higher total/HDL and HDL/LDL relative values.

**Table 6:** Dyslipidemia-related markers in HFD-induced obese rats compared to lean rats

Parameters	Groups	Min.	Max.	Mean ± SD	Percent change	*P* value^a^
CHOL	Lean	66.28	85.05	74.26 ± 6.97	100.00	
	HFD-obese	85.16	211.42	148.00 ± 48.24	199.29	0.026
HDL	Lean	42.22	65.73	54.95 ± 8.71	100.00	
	HFD-obese	22.59	67.32	51.45 ± 17.76	106.37	0.702
LDL	Lean	5.10	15.90	11.12 ± 3.89	100.00	
	HFD-obese	9.84	123.69	65.05 ± 43.59	484.98	0.075
TRIG	Lean	125.53	199.87	182.29 ± 31.89	100.00	
	HFD-obese	208.71	244.18	223.07 ± 14.77	122.37	0.032

^a^Refers to the significant difference between lean and HFD-induced obese rats.

**Table 7:** CHOL/HDL and HDL/LDL as atherosclerotic risk factors in HFD-induced obese rats compared to lean rats

Parameters	Groups	Min.	Max.	Mean ± SD	Percent change	*P* value a
CHOL∕HDL	Lean	1.15	1.75	1.52 ± 0.28	100.00	
	HFD-obese	2.15	3.77	3.02 ± 0.78	198.68	0.010
HDL∕LDL	Lean	2.65	5.91	4.61 ± 1.21	100.00	
	HFD-obese	0.52	2.29	1.15 ± 0.73	24.95	0.009

^a^Refers to the significant difference between lean and HFD-induced obese rats.

While lean rats recorded total/HDL and HDL/LDL ratios of 1.52 ± 0.28 and 4.62 ± 1.21, respectively, obese rats recorded much higher total/HDL ratios (3.02 ± 0.78) concomitant with much lower HDL/LDL ratios (1.15 ± 0.73). A total/HDL of 02 ± 0.78 was observed in obese rats.

**Figure 2:**
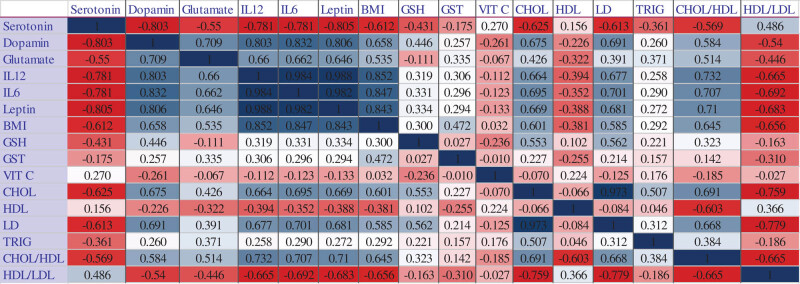
Heat map of Pearson’s correlations between all of the measured variables.

Figure 2 demonstrates the correlations between all of the measured markers, either in serum or in brain homogenates. It can easily be noticed that serotonin as a brain neurotransmitter was negatively correlated with dopamine and glutamate as brain-related variables. Serotonin also shows negative correlations with inflammatory cytokines (IL-12 and IL-6), obesity (leptin and BMI), antioxidant status (GSH), and dyslipidemia-related markers (cholesterol, triglycerides, total/HDL), respectively.

**Figure 3:**
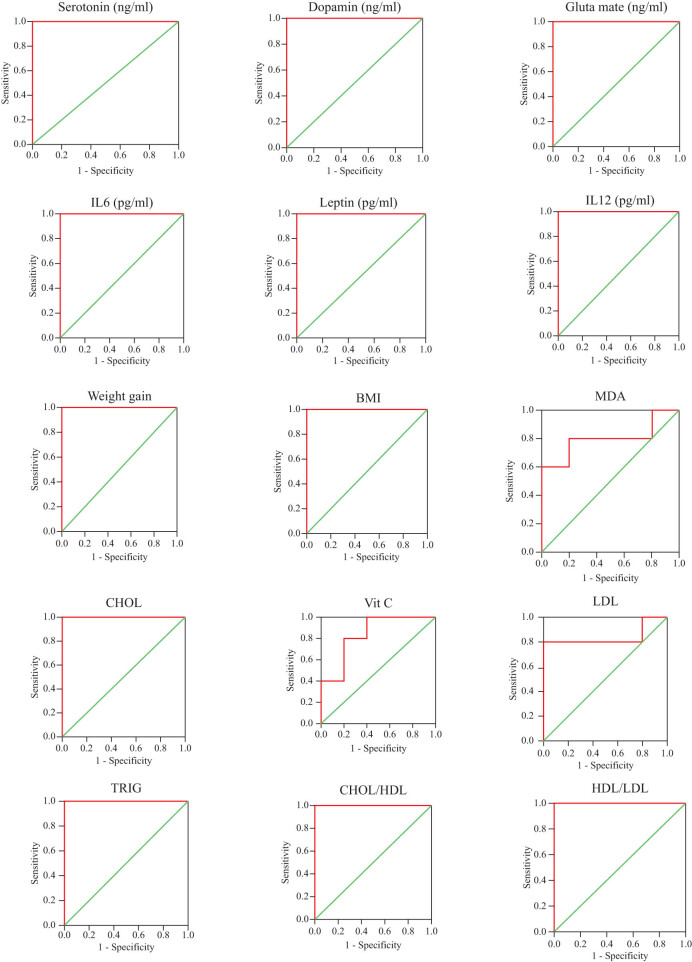
ROC curve analyses of all parameters for HFD-induced obese rats.

Table 8 and Figure 3 present the ROC curves of all of the measured variables in HFD-induced obese rats. AUCs, specificity, and sensitivity are clearly presented in the table.

**Table 8:** ROC curve analyses of all parameters for HFD-induced obese rats

Parameters	AUC	Cut-off value	Sensitivity %	Specificity %	*P* value	95% CI
Serotonin	1.000	55.000	100.0	100.0	0.009	1.000–1.000
Dopamine	1.000	60.500	100.0	100.0	0.009	1.000–1.000
Glutamate	1.000	49.500	100.0	100.0	0.009	1.000–1.000
IL12	1.000	91.910	100.0	100.0	0.009	1.000–1.000
IL6	1.000	180.995	100.0	100.0	0.009	1.000–1.000
Leptin	1.000	2.240	100.0	100.0	0.009	1.000–1.000
Weight gain	1.000	312.000	100.0	100.0	0.009	1.000–1.000
BMI	1.000	0.705	100.0	100.0	0.009	1.000–1.000
MDA	0.800	102.640	80.0	80.0	0.117	0.494–1.106
GSH	0.680	148.060	100.0	60.0	0.347	0.301–1.059
GST	0.600	0.911	40.0	100.0	0.602	0.219–0.981
Vitamin C	0.840	0.028	80.0	80.0	0.076	0.580–1.100
CHOL	1.000	85.105	100.0	100.0	0.009	1.000–1.000
HDL	0.560	58.545	60.0	80.0	0.754	0.168–0.952
LDL	0.840	29.425	80.0	100.0	0.076	0.544–1.136
TRIG	1.000	204.290	100.0	100.0	0.009	1.000–1.000
CHOL/HDL	1.000	1.950	100.0	100.0	0.009	1.000–1.000
HDL/LDL	1.000	2.470	100.0	100.0	0.009	1.000–1.000

Through the use of Pearson correlations, leptin displayed the highest (inverse) correlation to serotonin followed by IL-6 and IL-12. Dopamine and serotonin were inversely correlated, and IL-6, IL-12, and leptin were the most (positively) correlated markers to dopamine and glutamate. To test the predictive power of multivariate biomarker profiles, we used stepwise multiple regression, which showed that leptin is the sole statistically significant predictor of serotonin (*R* ^2^ = 0.648, *p* = 3.9185 × 10^−10^). For dopamine and glutamate, the statistically significant biomarker profiles were composed of IL-6 and GSH (*R* ^2^ = 0.725, *p* = 0.043457) for the former and IL-6, GSH, and triglycerides (*R* ^2^ = 0.617, *p* = 0.027508) for the latter. The coefficients shown in Table 9 indicate the dependent variable change for each independent variable unit increase.

**Table 9:** Predictive models of the dependent variables, serotonin, dopamine, and glutamate generated using multiple regression analysis of 13 independent variables

Dependent variable	*R* ^2^ of the model (multivariate biomarker profile)	Model significance	Independent variables	Coefficient	95% confidence interval	
					Lower boundary	Upper boundary
Serotonin	0.648	3.9185 × 10^−10^	Leptin	−12.861	−15.977	−9.745
Dopamine	0.725	0.043457	IL-6	0.239	0.181	0.296
			GSH	0.067	0.002	0.132
Glutamate	0.617	0.027508	IL-6	0.138	0.095	0.182
			GSH	−0.087	−0.136	−0.039
			Triglycerides	0.089	0.01	0.168

Table 10 demonstrates the bacterial plate count in different culture media for lean and HFD-induced obese rats. Data from the present study indicated that the dominant bacterial types in Group 1 (control group) in the first and second collections were gram-positive cocci, mainly *Streptococcus* coliforms. Gram-negative bacteria were either entirely absent or present in negligible numbers, and both *Clostridium* and *Bacteroides* were similarly absent in the control group. However, the opposite was true for Group 2, particularly following the HFD intake in the first and second collections, where gram-positive bacteria were overwhelmed by the overgrowth of gram-negative *Clostridium* and *Bacteroides*.

**Table 10:** Bacterial plate count in different culture media for lean and HFD-induced obese rats

Identified group strains/collection time	Gram (+ve) cocci (*Streptococcus*) (nutrient agar, and blood agar)	Gram (−ve) rods(*E. coli* or other coliforms) (MacConkey agar)	*Clostridium* sp. (CCFA agar plates)	*Bacteroides* sp.(BBE agar plates)
Lean first collection	100	1	0	0
	90	1	0	0
	95	0	0	0
Obese first collection	0	250	200	>300
	0	230	200	>300
	0	200	200	>300
			210	
Lean second collection	8	0	0	0
	6			
	7			
Obese second collection	0	>300	>300	200
	0	>300	>300	180
	0	250	280	190

## 4 Discussion

The recorded higher BMI in obese rats parallels the findings of Novelli et al. [24], who reported that BMI in obese rats usually has a value higher than 0.68 g/cm^2^. This observation is in good agreement with the previous work of Picklo et al. [25], which proved the obesogenic effect of a saturated lipid diet in an animal model.

It is well known that dopamine and serotonin play important roles in homeostatic signaling as neurotransmitters [26,27]. Numerous studies performed in humans and rodents, including those by Palmiter [28] and Halford et al. [29], show that experimental inhibition or stimulation of both transmitters is connected to differences in feeding behavior, the stimulus to eat, energy expenditure, and reward learning. Based on these as well as other observations, it can be hypothesized that changes in feeding behavior in individuals with obesity are a result of alterations in the central serotonin and dopamine systems [29,30,31].

The remarkably lower brain serotonin (5-HT) level in HFD-induced obese rats reported in the present study (Table 2) is supported by the recent work of van Galen et al. [31], in which a notable decrease in serotonin in obese subjects compared to overweight and lean controls using neuroimaging trials was reported. Their observations bolster the existing neurotransmitter theories of distressed feeding behavior in obesity. Apart from implications for mental health, 5-hydroxytryptamine (5-HT) receptors also play a significant role in obesity, which is usually accompanied by depression [32]. Interestingly, Noble and Kanoski [33] reported on the relationship between the obesogenic effects of a saturated HFD, as used in the current study, and hippocampal dysfunction, including increased neuroinflammation and neurogenesis and synaptic plasticity, which can give further support to our findings.

The significant increase in brain dopamine in obese rats (Table 2) can be explained by the fact that the dopamine system has a crucial role with regard to hyperphagia, high-energy diets, and obesity development [34,35].

The significant increase in brain glutamate reported in the present study is supported by the recent work of Fritz et al. [35], in which it was found that mice fed with an HFD have a focally extended excitatory postsynaptic current, likely due to lowered glutamate buffering (conversion to glutamine or gamma aminobutyric acid (GABA)) and/or blunted glutamate receptors (NMDA-R), with much higher glutamate signaling in the brain. In evaluating the synaptic function of obese animals within the dorsal striatum, they observed that obesity is linked to changes in glutamate transmission and greatly enhanced transmission of dopamine. These research results provide a novel insight into how high fat consumption affects neural mechanisms and the possible role of these mechanisms with regard to exaggerated nonhomeostatic eating.

The significant increases in IL-6 and IL-12 proinflammatory cytokines (Table 3) are consistent with the recent work of Yu et al. [36], in which IL-6 was much higher in the serum and brain of HFD-induced obese rats. In their study, hippocampal inflammatory responses were enhanced in obese rats, including the activation of TLR4 and NF-κB, the production of proinflammatory cytokines (IL-6), and the activation of microglia and astrocytes. In addition, hippocampal cell apoptosis and cognitive impairment were observed in the HFD-fed rats. This finding can be related to the remarkable alterations in serotonin, dopamine, and glutamate as markers of neuronal damage previously discussed in the present study (Table 2).

The reported significant increase in leptin in HFD-induced obese rats compared to that in lean rats (Table 4) is in good agreement with the previous work of Lin et al. [37], who showed that obesity induced by an HFD in C57 B1/6J mice may occur in three stages: an early response to the HFD due to exogenous leptin sensitivity, lowered food intake when leptin production increases and the brain remains sensitive to leptin, and finally, an elevated intake of food with reduced central sensitivity to leptin. This observation can also be supported by the most recent work of Mzhelskaya et al. [38], in which it was reported that in HFD-induced obesity, leptin loses its anorexigenic effect on neurons of the hypothalamus, consequently increasing appetite and fat mass accumulation.

The concentration of the serum MDA may be a useful indicator of oxidative stress. MDA is one of the final products in the peroxidation of polyunsaturated fatty acid by the cell. The concentration of MDA can be used as an indicator of cell or tissue damage due to increased lipid peroxidation activity. However, although the significant increase in BMI serves as a signal for obesity, impairment of neurotransmission, induction of inflammation, and leptogenesis, obese controls did not show a significant increase in MDA or decrease in GSH, GST, and vitamin C as antioxidants (Table 5). This result might be attributed to the addition of coconut oil as an inducer of obesity to the high saturated fat diet in our experiment. This prediction is supported by multiple studies that highlight the antioxidant effects of virgin coconut oil [39,40].

The ratio between total and HDL cholesterol is established as a useful lipid atherogenesis indicator, which reflects the transport of cholesterol within the arterial intima [41]. Based on the fact that total/HDL and HDL/LDL are both used as measures of risk factors of cardiovascular diseases, we suggest that the obese rats in the present study are at considerable risk (Tables 6 and 7). While lean rats recorded low ratios, obese rats recorded much higher total/HDL concomitant with much lower HDL/LDL ratios. A high total/HDL ratio in obese rats demonstrates a high atherogenic risk. This idea is supported by the work of Nam et al. [42], in which a total/HDL ratio of 2.1–3.6 was recorded as a risk factor for chronic heart disease.

LDL is responsible for the transport of cholesterol into peripheral tissues, while HDLs mediate an inverse transport of cholesterol [43]. The nonsignificant decrease in HDL in obese rats reported in the present study may be related to the antioxidant effect of the coconut oil component in our HFD.

In general, coconut oil ingestion is thought to increase HDL [44]. Lauric acid, which accounts for about 50% of the coconut oil content, is proposed to be the cornerstone of the pathway. Even though lauric acid is classified as a medium-chain fatty acid (MCFA), 70% of it gets transported as a long-chain fatty acid while 30% remains as an MCFA [45]. Thus, the transport of lauric acid happens in two ways in the body. Lauric acid that is transported to the liver functions as a substrate for the production of apoA1 and apoB, both of which further contribute to the production of low-density lipoprotein cholesterol (LDL-C) and high-density lipoprotein cholesterol (HDL-C) particles.

Research has shown that HDL, in contrast to LDL, may have an antithrombotic and antiatherogenic function [46] by protecting LDL from lipid peroxidation as well as reducing the harmful effect of oxidized LDL. Based on this finding, the nonsignificant decrease in HDL as an antiatherogenic molecule can be linked to the presence of coconut oil in the HFD [45].

The Pearson correlation coefficient is a statistical metric that measures the strength and direction of a linear relationship between two or more random variables [47]. The reported negative correlations between serotonin and inflammatory cytokines (IL-12 and IL-6), obesity (leptin and BMI), antioxidant status (GSH), and dyslipidemia-related markers (cholesterol, triglycerides, and total/HDL), respectively, can find support in the recent work of van Galen et al. [31], who highlighted that the neuroinflammation and dyslipidemia complications of obesity may be associated with decreased serotonergic signaling.

Additionally, dopamine and glutamate were positively correlated with neuroinflammatory cytokines (IL-12 and IL-6), leptin and BMI as markers of obesity, and cholesterol as an atherogenic marker (Figure 2). Again, this finding can be supported by the work of van Galen et al. [31], which reported on the decrease in dopamine receptors proportional to BMI in obese individuals.

Glutamate, the most dominant excitatory neurotransmitter in the brain, is involved in nearly all normal functions of the brain, including memory, learning, and cognition. Monosodium glutamate (MSG), also called sodium glutamate, is used as a flavor enhancer in the food industry. MSG gives a flavor that is impossible to achieve with other food additives [48,49,50,51]. The obtained positive correlations between brain glutamate as an excitatory neurotransmitter and most of the obesity-related markers in the current study can be supported through the recent work of Hernández Bautista et al. [52], in which an MSG-induced obesity model was associated with inflammation and impaired leptin levels.

The AUCs of the measured variables ascertained the effectiveness of the variables as predictive biomarkers for the development of synaptopathy as a complication of HFD-induced obesity (Table 8 and Figure 3). All variables recorded AUCs of one or near one with satisfactory specificity and sensitivity, showing excellent predictability, with the exception of GSH, GST, and HDL, which showed poor values, with AUCs between 0.5 and 0.7.

The multiple regression analysis presented in Table 9 ascertained the relationship between the three measured neurotransmitters and certain obesity-related markers. We were surprised to find that serotonin was greatly associated with leptin as an obesity-related hormone (*R* ^2^ of 0.648). This result is supported by the previous work of Yadav et al. [53], in which it was reported that leptin controls appetite and energy expenditure at least in part through the inhibition of serotonin synthesis and release from brainstem neurons. Moreover, Zou et al. [54] reported on the role of leptin in mood disorders, which are traditionally related to a lack of serotonin [55]. Table 9 also demonstrates the contributions of IL-6 and GSH as predictor markers of inflammatory and oxidative stress in the reported change of dopamine when used as a dependent variable in the multiple regression analysis (*R* ^2^ of 0.725). This result is in good agreement with other studies where obesity-related inflammatory and oxidative stress conditions have been repeatedly connected to alterations in reward circuitry and dopamine signaling [30,56]. Glutamate as a dependent variable was affected by IL-6, GSH, and triglycerides as respective inflammatory, oxidative, and dyslipidemia-related markers. This finding parallels the recent work of Hernández Bautista et al. [52], which illustrated the association between glutamate as an excitotoxic amino acid and obesity-related markers of inflammation, oxidative stress, and dyslipidemia. The reported elevated glutamate is in good agreement with the research findings, which strongly support the hypothesis that elevated levels of nutritional glutamate are etiologically important in obesity development in humans [57,58]. In their clinical trial, they were able to treat obesity by protecting the hypothalamic signaling cascade of leptin action with low to moderate affinity, noncompetitive glutamate NMDA-R antagonists that selectively block the GLU-gated Ca^2+^ ion channels. Additionally, an HFD disrupts the inhibitory GABAergic processes, leading to imbalanced inhibitory/excitatory neurotransmission in the frontal cortex and hippocampus of rats concomitant with abnormal feeding behavior [59]. This concept suggests that considerable efforts should be devoted to the development of weight-control medications that target the brain neurotransmitters that regulate food intake.

Data from the present study indicated that an HFD stimulated the overgrowth of *Bacteroides*, which is in agreement with other findings that highlighted how feeding mice an HFD led to a decrease in the abundance of *Firmicutes* from 72.1 to 34.5% and a significant increase in the *Bacteroides* population from 19.8 to 57.1% at the phyla level [60,61].

In conclusion, the present study illustrated that obesity-related inflammation, oxidative stress, and dyslipidemic processes, originating from either adipose tissue or the gut microbiota milieu, can spread to the brain, where they lead to considerable alterations in neurotransmitter metabolism and activity. Collectively, these alterations contribute to obesity-related neuropsychiatric complications.
